# Epigenetic signatures of internal migration in Italy

**DOI:** 10.1093/ije/dyu198

**Published:** 2014-10-15

**Authors:** Gianluca Campanella, Silvia Polidoro, Cornelia Di Gaetano, Giovanni Fiorito, Simonetta Guarrera, Vittorio Krogh, Domenico Palli, Salvatore Panico, Carlotta Sacerdote, Rosario Tumino, Paul Elliott, Giuseppe Matullo, Marc Chadeau-Hyam, Paolo Vineis

**Affiliations:** ^1^Department of Epidemiology and Biostatistics, Imperial College London, London, UK,; ^2^Human Genetics Foundation (HuGeF), Turin, Italy,; ^3^Department of Medical Sciences, University of Turin, Turin, Italy,; ^4^Fondazione IRCCS – Istituto Nazionale dei Tumori, Milan, Italy,; ^5^Istituto per lo Studio e la Prevenzione Oncologica (ISPO Toscana), Florence, Italy,; ^6^Department of Clinical Medicine and Surgery, University of Naples Federico II, Naples, Italy,; ^7^Piedmont Reference Centre for Epidemiology and Cancer Prevention (CPO Piemonte), Turin, Italy; ^8^Cancer Registry and Histopathology Unit, Azienda Ospedaliera ‘Civile – M. P. Arezzo’, Ragusa, Italy and; ^9^MRC-PHE Centre for Environment and Health, Imperial College London, London, UK

**Keywords:** Migration, DNA methylation, developmental origins of disease

## Abstract

**Background:** Observational studies have suggested that the risks of non-communicable diseases in voluntary migrants become similar to those in the host population after one or more generations, supporting the hypothesis that these diseases have a predominantly environmental (rather than inherited) origin. However, no study has been conducted thus far to identify alterations at the molecular level that might mediate these changes in disease risk after migration.

**Methods:** Using genome-wide DNA methylation profiles from more than 1000 Italian participants, we conducted an epigenome-wide association study (EWAS) to identify differences between south-to-north migrants and their origin (southern natives) and host (north-western natives) populations.

**Results:** We identified several differentially methylated CpG loci, in particular when comparing south-to-north migrants with north-western natives. We hypothesise that these alterations may underlie an adaptive response to exposure differentials that exist between origin and host populations.

**Conclusions:** Our study is the first large agnostic investigation of DNA methylation changes linked to migratory processes, and shows the potential of EWAS to investigate their biological effects.

**Key Messages**

- The risks of many non-communicable diseases in voluntary migrants become similar to those in the host population after one or more generations, but the involvement of alterations at the molecular level (such as DNA methylation) in this process is unclear.
- Using genome-wide DNA methylation profiles from more than 1000 Italian participants, we conducted an epigenome-wide association studies (EWAS) to identify differences between southern migrants to north-western Italy, and their origin and host populations.
- We identified several differentially methylated CpG loci, in particular when comparing south-to-north migrants with north-western natives.
- We hypothesize that these alterations may be part of an adaptive response to cope with the ‘mismatch’ between early life programming (due to perinatal exposures) and changes that occurred later in life as a result of migration.

## Introduction

Observational studies have contributed to consolidating the idea that the risks of many non-communicable diseases in voluntary migrants become similar to those in the host (native) population after one or more generations.^1–3^ For example, seminal studies have revealed a gradient of increasing incidence of coronary heart disease in Japanese men from Japan to Hawaii to California.^3^^,^^4^ These observations have been used to support the hypothesis that these diseases have a predominantly environmental origin (rather than inherited). Nonetheless, differences persist between native and migrant populations. For instance, migrants from non-western countries are more prone to cancers related to infections experienced in early life, and less likely to suffer from cancers commonly associated with a Westernized lifestyle.^5^

In this paper, we speculate that the observed health differentials might be mediated at the molecular level by changes in DNA methylation. In particular, we hypothesize that these changes are brought about by exposure differentials between the origin and host populations, and that they are instrumental in coping with the ‘mismatch’ between early-life programming (due to perinatal exposures) and changes in those same exposures that occurred later in life as a result of migration. This hypothesis is based on the concept that developmental history leaves its mark primarily through potentially reversible epigenetic changes.^6^ It is also supported by the observation that disease risks in migrants tend to increase with duration of residence in the host population, eventually becoming indistinguishable from those in natives.^3^ Among epigenetic changes, DNA methylation is thought to be relatively stable due to its heritability across cell generations, and yet flexible enough to allow differentiation into different cell types, as well as adaptation to stress and the external environment.^7–9^ In addition, DNA methylation plays a pivotal role in transcriptional repression and suppression of transcriptional noise,^10^ and is tightly linked to other epigenetic mechanisms such as histone modifications and chromatin remodelling.^11^^,^^12^ DNA methylation levels are associated with environmental and lifestyle exposures such as tobacco smoking,^13^ and altered DNA methylation patterns have also been implicated in many human diseases.^14^

Traditionally, epidemiological studies of migrants endeavoured to elucidate the relative contributions of genetic background, environment and their interaction.^15^^,^^16^ Most studies have focused on the effects of international migration, since risk factor differentials tend to be larger across countries. In this case, genetic differences between migrants and the host population may hinder the identification of migration-specific effects. Italy represents an interesting natural experiment, not only for its pronounced economic, social and environmental south-to-north gradient and the mass migration of labour that took place from the mid 1940s to the 1970s,^17^^,^^18^ but also for its relative genetic homogeneity (with the possible exception of Sardinia^19^).

Using genome-wide DNA methylation profiles obtained from prospectively collected peripheral blood samples from 1066 participants in the Italian component of the European Prospective Investigation into Cancer and Nutrition (EPIC-Italy),^20^ we present the first epigenome-wide association study (EWAS) to identify DNA methylation changes associated with voluntary south-to-north migration that occurred within Italy in the three decades after the end of WWII.

## Methods

### Study population and sample selection

All participants were recruited between 1993 and 1998 as part of EPIC-Italy.^20^ Detailed lifestyle and dietary information was collected at enrolment using self-administered questionnaires and a validated food frequency questionnaire,^21^ respectively. Anthropometric measurements were obtained at the inclusion visit, as were peripheral blood samples that were aliquoted and stored in liquid nitrogen on the day of collection.

A total of 1222 genome-wide DNA methylation profiles were acquired as part of three separate prospective case-control studies nested within EPIC-Italy on breast cancer (N = 332), colorectal cancer (N = 338) and myocardial infarction (EPICOR,^22^ N = 552). Eight profiles were excluded because of unsatisfactory technical quality. A single profile was retained, on the basis of technical quality, for participants included in more than one study, leaving a total of 1170 unique profiled participants. Within each study, participants who developed the relevant condition less than 1 year after blood draw (N = 46), or who developed any kind of haematological malignancy at any time after enrolment (N = 4), were excluded; all remaining subjects were considered healthy at inception. A total of 23 participants were excluded because of incomplete dietary or lifestyle information. To minimize confounding by genetic factors, participants born outside Italy (N = 18) or in the insular region of Sardinia (N = 18) were also excluded. The remaining 1061 participants were categorized as follows:

- south-to-north migrants (N = 190), recruited in Turin (N = 148) or Varese (N = 42), and born in any southern Italian region;
- southern natives (origin population, N = 123), recruited by the two southern Italian EPIC centres of Naples (N = 40) and Ragusa (N = 83), and born in any southern Italian region;
- north-western natives (host population, N = 543), recruited by the two north-western Italian EPIC centres of Turin (N = 317) and Varese (N = 226), and born in any north-western Italian region.

A total of 205 participants did not fall into any of the above categories, and were excluded from subsequent analyses. Detailed information on the 856 participants included in the study is summarized in Table 1.

**Table 1.** Characteristics of participants included in the study. Counts and percentages are reported for categorical variables, and medians and ranges for continuous variables

	South-to-north migrants	Southern natives	North-western natives
N	190	123	543
Men	115 (60.5%)	62 (50.4%)	232 (42.7%)
Women	75 (39.5%)	61 (49.6%)	311 (57.3%)
Age (years)	51.2 (35.8 to 66.0)	52.7 (34.7 to 67.9)	54.4 (35.2 to 72.0)
Smoking status			
Never	64 (33.7%)	41 (33.3%)	248 (45.7%)
Current	65 (34.2%)	40 (32.5%)	151 (27.8%)
Former	61 (32.1%)	42 (34.1%)	144 (26.5%)
Physical activity			
1	30 (15.8%)	28 (22.8%)	116 (21.4%)
2	67 (35.3%)	36 (29.3%)	148 (27.3%)
3	67 (35.3%)	51 (41.5%)	251 (46.2%)
4	26 (13.7%)	8 (6.5%)	28 (5.2%)
5	0 (0%)	0 (0%)	0 (0%)
Energy (kcal)	2143.5 (487.7 to 5225.3)	2375.9 (1100.7 to 5476.9)	2184.0 (722.7 to 5309.2)
Protein (g)	85.5 (26.5 to 189.6)	94.1 (39.5 to 190.8)	89.7 (24.4 to 209.3)
From animal sources	53.9 (17.2 to 132.0)	51.3 (12.5 to 103.3)	58.2 (14.3 to 167.8)
From vegetable sources	29.3 (5.6 to 92.0)	40.0 (13.8 to 124.9)	26.3 (5.5 to 70.3)
Fat (g)	77.6 (21.0 to 199.0)	85.9 (36.3 to 178.4)	83.5 (18.9 to 227.7)
From animal sources	41.6 (14.5 to 108.5)	41.9 (12.1 to 105.1)	47.8 (6.4 to 174.6)
From vegetable sources	36.0 (5.6 to 90.5)	42.0 (12.5 to 91.6)	35.0 (4.4 to 91.0)
Cholesterol (mg)	326.7 (77.4 to 772.9)	288.4 (72.9 to 692.5)	358.0 (72.4 to 1002.5)
Available carbohydrates (g)	259.1 (50.8 to 685.9)	320.0 (120.3 to 780.5)	249.2 (48.9 to 760.6)
Soluble carbohydrates	93.6 (15.8 to 284.1)	95.5 (39.0 to 253.0)	98.5 (28.1 to 384.2)
Starch	158.5 (33.7 to 571.6)	209.2 (55.8 to 655.1)	144.0 (5.1 to 460.3)
Fibre (g)	21.0 (3.6 to 51.5)	33.4 (10.5 to 131.2)	19.5 (5.3 to 70.5)
Alcohol (g)	11.5 (0.0 to 90.9)	1.6 (0.0 to 50.4)	11.2 (0.0 to 105.4)
Vitamins			
Vitamin A (µg RE)	944.8 (167.1 to 3467.5)	898.5 (260.1 to 5092.5)	1031.7 (175.4 to 6716.7)
Vitamin B_1_ (mg)	0.96 (0.24 to 2.12)	1.22 (0.40 to 4.40)	0.98 (0.33 to 2.18)
Vitamin B_2_ (mg)	1.46 (0.30 to 3.12)	1.58 (0.57 to 3.19)	1.58 (0.43 to 4.19)
Vitamin B_3_ (mg)	17.8 (5.2 to 39.6)	21.9 (7.8 to 63.9)	17.8 (4.9 to 39.5)
Vitamin B_6_ (mg)	1.84 (0.45 to 4.40)	2.28 (0.85 to 5.75)	1.87 (0.46 to 4.12)
Folic acid (µg)	269.8 (48.3 to 775.7)	335.0 (124.4 to 835.0)	261.0 (50.8 to 673.8)
Vitamin C (mg)	127.5 (16.9 to 413.5)	153.2 (63.4 to 672.7)	121.5 (5.9 to 888.5)
Vitamin D (µg)	1.24 (0.13 to 5.85)	1.05 (0.04 to 6.30)	1.34 (0.08 to 12.45)
Vitamin E (mg)	7.65 (1.90 to 24.18)	9.08 (3.78 to 20.04)	7.69 (1.50 to 22.60)
Minerals			
Calcium (mg)	919.9 (279.6 to 2956.3)	834.3 (239.0 to 2050.2)	1020.1 (201.7 to 3833.4)
Iron (mg)	13.8 (2.9 to 35.0)	15.1 (5.8 to 42.9)	13.8 (4.8 to 31.9)
Phosphorus (mg)	1342.6 (382.9 to 2945.3)	1558.9 (576.4 to 3816.1)	1413.4 (487.0 to 3393.8)
Potassium (mg)	3207.2 (690.1 to 7023.0)	3564.4 (1414.5 to 8445.0)	3271.4 (982.1 to 8882.6)
Sodium (mg)	2164.9 (359.2 to 9837.6)	2365.8 (729.8 to 8879.0)	2227.3 (728.5 to 8636.5)
Zinc (mg)	11.7 (4.0 to 26.1)	14.2 (4.4 to 42.9)	12.4 (3.6 to 32.8)

### Laboratory analyses

Genome-wide DNA methylation analyses using the Illumina Infinium HumanMethylation450 (HM450) platform were carried out at the Human Genetics Foundation (Turin, Italy) according to manufacturers' protocols. Buffy coats stored in liquid nitrogen were thawed, and genomic DNA was extracted using the QIAGEN QIAsymphony DNA Midi Kit; 500 ng of DNA were bisulphite-converted using the Zymo Research EZ-96 DNA Methylation-Gold™ Kit, and hybridized to Illumina Infinium HumanMethylation450 BeadChips. These were subsequently scanned using the Illumina HiScanSQ system, and sample quality was assessed using control probes present on the microarrays. Finally, raw intensity data were exported from Illumina GenomeStudio (version 2011.1). Data pre-processing was carried out using in-house software written for the R statistical computing environment. For each sample and each probe, measurements were set to missing if obtained by averaging intensities over less than three beads, or if averaged intensities were below detection thresholds estimated from negative control probes. Background subtraction and dye bias correction (for probes using the Infinium II design) were also performed. The subset of 470 870 probes targeting autosomal CpG loci was selected for further analyses. DNA methylation levels at each locus were expressed as the ratio of intensities arising from methylated cytosines over total intensities.

### Statistical analyses

An EWAS was conducted to compare south-to-north migrants with their origin (southern natives) and host (north-western natives) populations, with the objective of characterizing epigenetic adaptation processes subsequent to migration to north-western Italy. For each probe, DNA methylation levels were modelled as dependent variable in a generalized linear model with beta-distributed response using the parameterization of Ferrari and Cribari-Neto.^23^ All models were adjusted for microarray (N = 102) and position on the microarray (N = 12), sex, and case-control status (separately for cancers and myocardial infarction). In place of age at recruitment, models were also adjusted for two continuous variables representing the time to birth and to recruitment of each participant (computed from an arbitrary reference date). Since the difference between these two quantities equals age at recruitment for any choice of reference date, this approach grants an additional degree of freedom to account for possible differences in migration behaviour associated with birth date. The effect of dietary and lifestyle factors, which are radically different in southern regions,^24^ was investigated using a second set of models additionally adjusted for 25 dietary variables (total energy intake, protein from animal and vegetable sources, fat from animal and vegetable sources, cholesterol, soluble carbohydrates, starch, fibre, alcohol and vitamins and minerals as listed in Table 1), smoking status and level of physical activity (categorical variable). To prevent inclusion of highly correlated variables and reduce the number of estimated regression coefficients, dietary variables were subjected to principal component analysis (PCA), and the first 16 principal components (explaining more than 99% of the variance) were included in the models. Multiple comparisons were accounted for by considering a Bonferroni-corrected significance threshold α = 0.05/470,870 ≈ 1.1 × 10^−7^, ensuring a stringent control of the family-wise error rate at level 5%. Candidate CpG loci were additionally filtered as follows. First, probe sequences were aligned to the reference human genome using Bowtie 2^25^ to assess the potential to cross-hybridize to multiple genomic locations, thus affecting DNA methylation measurements.^26^ CpG loci targeted by cross-hybridizing probes (defined as those lacking unique genome alignments, with up to three base mismatches) were excluded from further consideration. Second, potential sources of genetic confounding and context disruption for DNA methylation (such as polymorphisms at the CpG locus) were identified by retrieving known genetic variations and computing the corresponding minor allele frequencies (MAFs) in the European population, based on publicly available data generated by the 1000 Genomes project.^27^ As a precautionary measure, CpG loci found within 100 base pairs (bp) of non-rare variants (MAF greater than 1%) were removed from the list of candidates.

## Results

**Figure 1.**
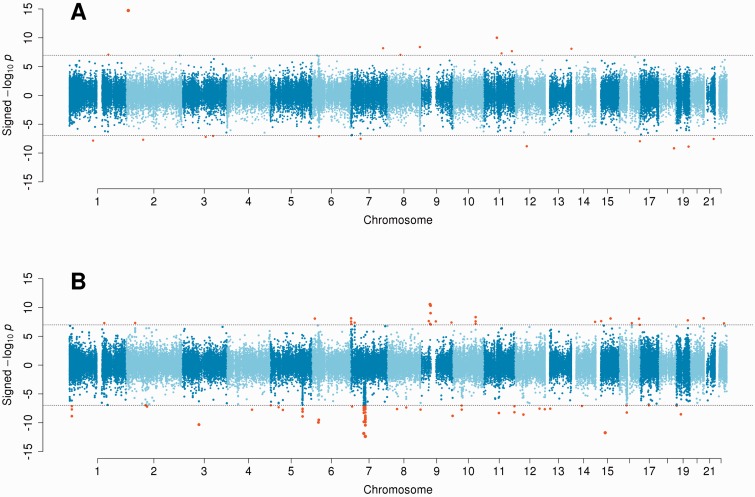
Signed Manhattan plot for the EWAS comparing south-to-north migrants with: (A) the origin population (southern natives); (B) the host population (north-western natives).

As illustrated in Figure 1A, the EWAS identified 20 differentially methylated CpG loci in south-to-north migrants with respect to the origin population (southern natives). Two probe sequences were ambiguously aligned to the reference human genome, and genetic variations were found in the vicinity of nine candidate CpG loci. A total of nine CpG loci were left for further consideration, of which none survived the adjustment for dietary and lifestyle factors (Supplementary Table 1, available as Supplementary data at *IJE* online).

**Figure 2.**
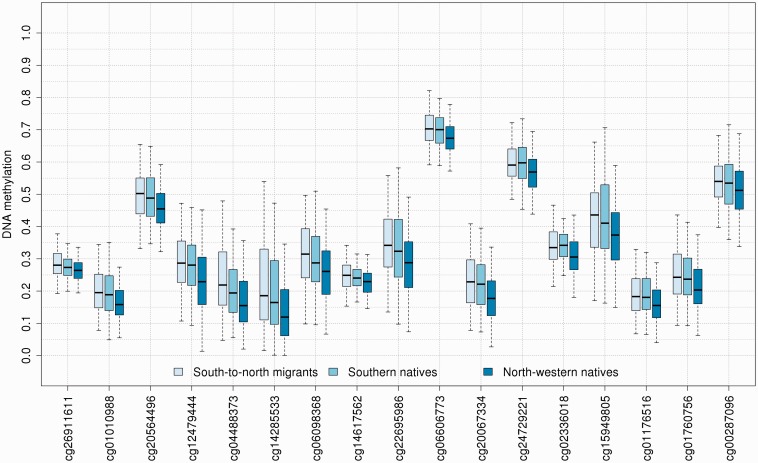
Box-and-whisker plot across groups for CpG loci identified by the EWAS in the pericentric region on the long arm of chromosome 7.

**Figure 3.**
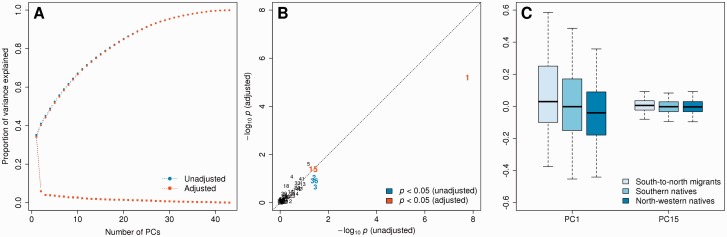
PCA of DNA methylation levels assayed in the pericentric region on the long arm of chromosome 7: (A) scree plot; (B) Manhattan plot for the association of PCs with migrant status (Kruskal–Wallis rank sum test); (C) box-and-whisker plot for PCs associated with migrant status (*P* < 0.05) after adjustment for dietary and lifestyle factors.

Comparison of south-to-north migrants with respect to the host population (north-western natives) revealed 91 differentially methylated CpG loci (Figure 1B). After removal of 23 candidates whose associated probe sequences could not be uniquely aligned to the reference human genome, and of 33 candidates in the proximity of non-rare genetic variations, 35 CpG loci were left for further consideration, and 22 survived the adjustment for dietary and lifestyle factors. Of these, 17 were found to be relatively hypermethylated in south-to-north migrants, and seven were found in the pericentric region on the long arm of chromosome 7 (from the centromere to 6.37 × 10^7^ bp). These loci exhibited a consistent decreasing gradient from south-to-north migrants to southern natives to north-western natives (Figure 2). They were also flanked by several other loci that shared the same direction of association. This region was additionally characterized by PCA of DNA methylation measurements at 43 enclosed CpG loci assayed by the HM450 platform (filtered according to the criteria described above), before and after adjustment for dietary and lifestyle factors. Irrespective of adjustment, the first principal component explained approximately 35% of the variance (Figure 3A), and was the only component explaining more than 10% of the variance. The association of each principal component with migratory status was formally assessed using Kruskal–Wallis rank sum tests. Results were comparable before and after adjustment; however, the second, third and 36th principal components lost statistical significance after adjustment (Figure 3B). The first principal component was consistently associated with migratory status (*P*-values 1.71 × 10^−8^ and 6.28 × 10^−6^ before and after adjustment, respectively), as was the 15th (*P*-values 0.043 and 0.041, respectively). Scores exhibited a decreasing gradient similar to that observed in Figure 2, albeit less markedly for the 15th principal component (Figure 3C).

## Discussion

To our knowledge, this is the first EWAS to examine DNA methylation changes in voluntary migrants. The gamut of alterations observed in south-to-north migrants offers evidence that important environmental and lifestyle changes may induce molecular adaptation mechanisms to stressors that are inheritable across cell divisions. Some of the differences are evident even after adjustment for dietary and lifestyle factors, suggesting that these DNA methylation changes are not merely ascribable to behaviour modification following migration. Intriguingly, we found DNA methylation changes in south-to-north migrants compared to the host population at several CpG loci located on a large pericentric region on the long arm of chromosome 7. Pericentric regions have long been thought to be transcriptionally inert, but recent evidence suggests that pericentric and centromeric transcripts play an important role in preserving genome stability.^28^ Additionally, transcription of pericentric satellites appears to be a general cellular response to external stressors including heat shock, ultraviolet radiation and oxidative stress.^29^ In this light, it appears that molecular consequences of migration may not be limited to specific genes, but may act at a higher complexity level, for example on gene regulatory networks. The gradient observed in Figure 2 may thus epitomize an adaptive mechanism to cope with the ‘mismatch’ between early-life programming and exposure changes in later life: before migration, south-to-north migrants and southern natives share common environmental factors that affect DNA methylation patterns and (possibly) differentiate them from northern natives; the amplified response observed after migration might therefore be a consequence of relative abundance or lack of these factors in the host population. Such factors could include, for example, vitamin D (in relation to more limited sun exposure in northern Italy), other vitamins contained in food, occupational and environmental exposure to pollutants and even exposure to different infectious agents (with some viruses, for example the hepatitis B virus, being more prevalent in southern populations). This would not only explain the observed DNA methylation gradient, but it would also be consistent with the ‘developmental origins of disease’ hypothesis,^30^^,^^31^ and with current understanding of the role of perinatal exposures in health and disease.

The main strengths of our study are its sample size and the relative genetic homogeneity of its participants (all born in Italy), which limits the potential for genetic confounding. Its main limitation is the lack of information regarding the time of migration, from which age at migration and duration of stay could be computed and accounted for. Nevertheless, absence of this information is more likely to dilute any observable effect on DNA methylation, rather than lead to false-positive results. The biological interpretation of our results could be enhanced were genome-wide gene expression data available for the same subjects. These would allow us to establish whether the observed DNA methylation changes are associated with gene expression and its regulation, and would thus provide a much deeper understanding of how migration exerts its biological effects at different cellular complexity levels. Despite these limitations, we think this work exemplifies the promising potential of EWAS approaches to elucidate complex and subtle effects of migration at the population level.

## Supplementary Data

Supplementary data are available at *IJE* online.

## Funding

This work was supported by the 7th European Framework Programme (FP7), grant agreement 308610 (Exposomics) to P.V. EPIC-Italy was financially supported by the Italian Association for Cancer Research (AIRC). Genome-wide DNA methylation profiling of EPIC-Italy samples was financially supported by the Human Genetics Foundation and Compagnia di San Paolo (Turin, Italy). G.C. receives a Doctoral Prize studentship awarded by the Engineering and Physical Sciences Research Council (EPSRC). P.E. is a National Institute for Health Research (NIHR) senior investigator and acknowledges support from the NIHR Biomedical Research Centre at Imperial College Healthcare NHS Trust and Imperial College London. He is supported by the Medical Research Council and Public Health England as part of joint funding for the MRC-PHE Centre for Environment and Health.

## Supplementary Material

Supplementary Data
