# Supplementary material for: Epigenetic signatures of internal migration in Italy
Source: Int J Epidemiol. 2014 Oct 15;44(4):1442–9. doi: 10.1093/ije/dyu198 (PMC4588856; doi:10.1093/ije/dyu198)
Supplement: Supplementary Data [file supp_44_4_1442__index.html]

Epigenetic signatures of internal migration in Italy — Epigenetic signatures of internal migration in Italy — Epigenetic signatures of internal migration in Italy — Supplementary Data 

# Epigenetic signatures of internal migration in Italy

## Supplementary Data

files

**Files in this Data Supplement:**

- Supplementary Data - xlsx file
